# Lower incidence of contrast-induced nephropathy in patients undergoing fluorescent angiography

**DOI:** 10.1186/s12886-017-0440-4

**Published:** 2017-04-19

**Authors:** Ji Hwan Lee, Byunghoon Chung, Sung Chul Lee, Sung Soo Kim, Hyoung Jun Koh, Christopher Seungkyu Lee

**Affiliations:** 1 The Institute of Vision Research, Department of Ophthalmology, Yonsei University College of Medicine, Severance Hospital, Yonsei-ro 50-1 Sodaemun-gu, Seoul, 03722 Republic of Korea; 2 Mungyeong City Public Health Center, Mungyeong, Republic of Korea

**Keywords:** Acute kidney injury, Contrast media, Fluorescein angiography

## Abstract

**Background:**

To evaluate the incidence and risk factors of contrast-induced nephropathy (CIN) in patients undergoing fluorescein angiography (FA).

**Methods:**

One hundred sixty patients who underwent FA as a part of ophthalmic examination and had serum creatinine (SCr) results within 24 h before FA and within 72 h after FA between 2005 and 2013 at a tertiary medical center were included. According to baseline SCr levels, the subjects were divided into low-risk group (<1.5 mg/dL), intermediate-risk group (1.5–2.0 mg/dL), and high-risk group (>2.0 mg/dL) for CIN development. The CIN incidence, and changes in renal function defined by SCr levels and estimated glomerular filtration rate (eGFR) were evaluated. Demographics and comorbidities were analyzed to investigate an association with CIN development.

**Results:**

Of 160 patients, 91 were males (56.9%). The mean age was 52.46 ± 17.81 years. Two (1.3%) patients developed CIN after FA, whose SCr levels returned to normal within 10 days without hemodialysis. Overall, there were no changes before and after FA in SCr level (1.52 ± 1.31 mg/dL vs. 1.51 ± 1.28 mg/dL, respectively; *p* = 0.93) and eGFR (67.02 ± 36.62 mL/min/1.73 m^2^ vs. 66.41 ± 36.54 mL/min/1.73 m^2^, respectively; *p* = 0.54). SCr level and eGFR remained unchanged after FA in low-risk and intermediate-risk groups. In high-risk group, eGFR remined unchanged, but SCr level decreased after FA (from 3.64 ± 1.59 mg/dL to 3.53 ± 1.60 mg/dL; *p* = 0.04). Basline SCr and cormorbidities did not predict CIN development.

**Conclusions:**

Acute renal function deterioration was not evident in patients undergoing FA regardless of baseline renal function and comorbidities.

## Background

Fundus fluorescein angiography (FA) is a crucial imaging tool in the diagnosis and management of various retinal diseases including diabetic retinopathy, age-related macular degeneration, and uveitis. Adverse reactions are usually mild and transient, but may include serious reactions such as anaphylaxis, seizures, and death [1–6]. Sodium fluorescein (C_20_H_12_O_5_Na, molecular weight 376.27 g/mol), a noniodinated contrast media used in FA is largely eliminated by the kidneys within 24 h, [7] so patients with renal insufficiency may be at increased risk of nephrotoxicity after FA. Common practice may dictate the avoidance of FA in patients with renal insufficiency, but patients who require FA imaging for conditions such as diabetic retinopathy often have concurrent kidney problems [8, 9].

Contrast-induced nephropathy (CIN) is a form of acute kidney injury defined by acute elevation of serum creatinine (SCr), generally within 72 h after the injection of contrast medium. CIN has been known to increase the cost of medical care, duration of hospital stay, and risk of serious long-term adverse events such as permanent renal impairment, myocardial infarction, pulmonary edema, stroke, and death [10]. CIN has received increasing attention in patients undergoing percutaneous coronary angiography or contrast-enhanced computed tomography (CT) scans [10, 11]. Known risk factors for CIN include older age, diabetes mellitus (DM), pre-existing renal failure, heart failure, higher volumes of injected contrast media, dehydration, and concurrent nephrotoxic drugs [11]. However, CIN in the ophthalmological field has not been investigated, yet; PubMed search (keywords: contrast-induced nephropathy and fluorescein angiography) showed no report on CIN associated with FA. We conducted a retrospective study to investigate whether FA is associated with the deterioration of acute renal function and determine the CIN occurrece rate and associated risk factors.

## Methods

Medical records for all patients seen at the Department of Ophthalmology, Severance Hospital, College of Medicine, Yonsei University between November 2005 and October 2013 were screened retrospectively. This study was approved by the Institutional Review Board of Severance hospital (IRB No.4-2014-0799) and was conducted in accordance with the Declaration of Helsinki.

Patients were included if they had: (a) undergone FA between November 1, 2005 and October 31, 2013 (b) baseline SCr results within 24 h before FA (c) SCr results within 72 h after FA. Exclusion criteria included pre-existing medical conditions requiring renal dialysis and having undergone concurrent CT scans or percutaneous angiography using contrast media within two weeks before FA [12]. If there were two or more post-FA SCr levels available within 72 h after FA, the highest SCr level was selected.

Predisposing medical/ophthalmologic conditions that are known to be risk factors for CIN were identified using International Classification of Diseases, Tenth Edition, Clinical Modification (ICD-10), which included diabetic nephropathy (DMN), congestive heart failure (HF), and chronic kidney disease (CKD). The diagnosis of CKD was confirmed using conventional criteria (estimated glomerular filtration rate (eGFR) < 60 mL/min/1.72 m^2^) [11, 13–15]. eGFR was calculated using the Modification of Diet in Renal Disease formula and included serum albumin and blood urea nitrogen levels.

Patients were stratified according to baseline SCr level as follows: low-risk group (SCr < 1.5 mg/dL), intermediate-risk group (SCr 1.5–2.0 mg/dL) and high-risk group (SCr > 2.0 mg/dL) [16–18].

CIN was defined as a rise in SCr level of 0.5 mg/dL or greater or as a 25% increase over baseline level within 72 h after FA [19, 20]. In the present study, a rise in SCr level of 0.5 mg/dL was chosen as a threshold value over 0.3 mg/dL, which was recently proposed by the Acute Kidney Injury Network, because higher value is potentially more specific, less likely to yield false-positive results from cumulative biologic and assay variability, and more commonly used as a definition of CIN in current medical practice [17, 19, 21, 22].

All patients received 500 mg of sodium fluorescein (Novartis, Basel, Switzerland) intravenously through an antecubital vein in 5–8 s. Statistical analyses were performed using R software (version 2.15, R Foundation for Statistical Computing, Vienna, Austria). A paired student’s *t* test and Wilcoxon signed rank test were used to compare SCr between various groups; all *p* values of <0.05 were considered statistically significant.

## Results

A total of 160 patients (91 male, 69 female) were lincluded. The mean age was 52.46 ± 17.81 years. The demographic information and clinical findings are presented in Table 1. Post-FA SCr and eGFR were obtained within 24 h after FA in 96 patients (60.0%), between 24 and 48 h in 42 patients (26.3%), and between 48 and 72 h in 22 patients (13.7%). Baseline SCr level was 1.52 ± 1.31 mg/dL, and baseline eGFR was 67.02 ± 36.62 mL/min/1.73 m^2^. There were no changes in SCr level and eGFR after FA.

**Table 1:** Demographic Information, SCr Levels, and eGFR for Patients with Presumed Risk of Developing Acute Kidney Injury

Group	Total	Low risk	Intermediate risk	High risk
Number	160	113	15	32
Age	52.46 ± 17.81	51.76 ± 18.84	54.87 ± 14.28	53.78 ± 15.64
Sex (M/F)	91/69	55/58	7/8	29/3
Pre-SCr (mg/dL)	1.52 ± 1.31	0.90 ± 0.27	1.60 ± 0.11	3.64 ± 1.59
Post-SCr (mg/dL)	1.51 ± 1.28	0.93 ± 0.32	1.63 ± 0.26	3.53 ± 1.60
*p* value	0.93	0.13	0.65	0.04*
Pre-eGFR (mL/min/1.73 m^2^)	67.02 ± 36.62	84.47 ± 29.71	41.81 ± 6.77	21.56 ± 8.81
Post-eGFR (mL/min/1.73 m^2^)	66.41 ± 36.54	83.23 ± 30.69	41.36 ± 6.76	22.93 ± 10.47
*p* value	0.54	0.37	0.81	0.08

Values are presented as mean ± SD (standard deviation)

*p value < 0.05

When patients were grouped according to their baseline SCr levels, there were no changes in SCr level and eGRF after FA in low-risk group and intermediate-risk group. In high-risk group, SCr level decreased after FA, while there was no change in eGFR (Table 1). SCr levels and eGFR remained unchanged after FA when they were analyzed according to coexisting medical/ophthalmologic conditions that are known risk factors of CIN (Table 2). SCr levels and eGFR did not change after FA regardless of whether patients were inpatients or outpatients (Table 3).

**Table 2:** Changes in Serum Creatinine and eGFR According to Coexisting Conditions

Group	DM with nephropathy	DM without nephropathy	CKD	HF
Number	15	61	27	7
Age				
median	55	59	53	63
interquartile range	46–66	50–69	42–64	46–68
Sex (M/F)	7/8	35/26	22/5	3/4
Pre-SCr (mg/dL)				
median	1.60	1.16	2.84	1.33
interquartile range	1.21–3.20	0.89–1.67	1.50–4.99	0.77–1.49
Post-SCr (mg/dL)				
median	1.70	1.20	2.74	1.31
interquartile range	1.27–3.20	0.89–1.74	1.42–4.58	0.84–1.67
*p* value	1.000	0.131	0.115	0.735
Pre-eGFR (mL/min/1.73 m^2^)				
median	38.68	55.76	24.00	54.94
interquartile range	17.28–49.36	35.84–72.76	13.00–39.00	38.68–79.36
Post-eGFR (mL/min/1.73 m^2^)				
median	36.32	55.88	26.00	58.83
interquartile range	20.00–47.00	31.91–72.68	15.21–47.00	33.91–71.78
*p* value	0.398	0.170	0.679	0.310

**Table 3:** Changes in Serum Creatinine and eGFR of Inpatients and Outpatients

	Inpatients	Outpatients
Number	149	11
Pre-SCr (mg/dL)		
median	1.10	0.84
interquartile range	0.80–1.57	0.80–1.00
Post-SCr (mg/dL)		
median	1.07	0.81
interquartile range	0.80–1.65	0.70–2.08
*p*-value	0.993	0.656
Pre-eGFR (mL/min/1.73 m^2^)		
median	63.99	79.87
interquartile range	38.52–89.00	51.54–104.06
Post-eGFR (mL/min/1.73 m^2^)		
median	61.28	82.37
interquartile range	38.64–88.00	32.55–101.53
*p*-value	0.673	0.859

Of 160 patients, 2 (1.3%) patients developed CIN, 1 in the low-risk group and 1 in the intermediate-risk group (Table 4). Patient number 1 was a 53-year-old male with underlying hypertension. He was admitted to the ophthalmology department due to bilateral acute syphilitic posterior placoid chorioretinitis. His SCr level increased from 0.84 mg/dL to 2.08 mg/dL 24 h after FA; however, SCr level decreased rapidly and was normalized to baseline level (0.81 mg/dL) within 3 days without treatment. Patient number 2 was a 62-year-old diabetic male. He was admitted due to sphenoethmoidal aspergillosis and was evaluated in ophthalmological department for decreased visual acuity. His SCr level increased by 0.8 mg/dL after FA (1.5 mg/dL to 2.3 mg/dL) within 72 h. Nephrologist suspected amphotericin-related nephropathy and changed systemic medication from amphotericin B to voriconazole. His SCr level decreased to baseline level (1.7 mg/dL) within 10 days.

**Table 4:** Demographic Information, SCr Levels, and eGFR prior and Subsequent to fluorescein angiography

Patient No.	Age	Sex	Pre-SCr (mg/dL)	Post-SCr (mg/dL)	Change in SCr (mg/dL)	Pre-eGFR (mL/min/1.73 m^2^)	Post-eGFR (mL/min/1.73 m^2^)
1	53	M	0.84	2.08	1.24	90.0	34.0
2	62	M	1.50	2.30	0.80	50.5	30.8

SCr: serum creatinine

eGFR: estimated glomerular filtration rate

## Discussion

There were no significant changes in SCr levels and eGFR after FA in patients with various baseline SCr levels. In the high-risk group, SCr levels actually decreased following FA, but this change is clinically insignificant, as a reduction in SCr after FA may not indicate improvement in renal function. Female gender has been identified as an independent risk factor for CIN [23]. The relatively lower portion of female patients in high-risk group than intermediate and low-risk groups (9.4% Vs 46.7% and 48.7%) may have affected results in the high-risk group after FA. We analyzed eGFR in addition to SCr, as SCr values can vary with age, muscle mass, and sex. Both SCr and eGFR remained unchanged after FA regardless of baseline SCr levels, coexisting comorbidities, and inpatient/outpatient status.

CIN has been investigated mainly in the field of cardiology and radiology. CIN occurs in 5.2–33% of patients undergoing cardiac catheterization [24] and 4–12% of patients undergoing a contrast-enhanced CT scan [20, 25–28]. The rate of 1.3% found in the present study appeared to be significantly lower compared to CIN associated with cardiac catheterization and contrast CT scans. Intra-arterial administration of contrast media, higher dosage, and the invasive nature of coronary angiography may explain higher CIN rate in cardiac catheterization. In FA, fluorescein is injected intravenously in lower doses.

Another reason for lower CIN rate in FA may be the difference in the nature of contrast media used. A noniodinated fluorescein with lower molecular weight is used in FA, whereas iodinated contrast agents that have are commonly used in CT scans, cardiac angiography, and other conventional radiographic imaging techniques [29]. Although the exact mechanisms underlying contrast media nephrotoxicity are unknown, increased vasoconstrictive forces, decreased local prostaglandin- and nitric oxide-mediated vasodilatation, toxic effect of free radicals on renal tubular cells, increased intratubular pressure secondary to contrast-induced diuresis, increased urinary viscosity, and tubular obstruction are thought to play roles in the development of CIN [30]. Relatively high osmolality and viscosity of iodinated contrast media may be related to these pathogenic processes [31]. However, whether contrast media itself plays a causal role in developing acute kidney injury is controversial [20]. In a recent study, the incidence of CIN in patients undergoing contrast-enhanced CT did not differ significantly from that in patients undergoing nonenhanced CT [32].

Pre-existing renal disease is one of the most important risk factor for CIN [12]. In the present study, risk stratification according to baseline SCr levels did not predict CIN development. Patients with co-existing diabetic nephropathy and chronic kidney disease showed no change in SCr and eGFR after FA as well. A conventional risk-stratification scheme based on SCr levels for renal function may not be an adequate method for patients undergoing FA. It raises questions as to whether FA is contraindicated in patients with elevated SCr levels or poor eGFR, and nephrology consultation before FA is an absolute necessity. In agreement with our finding, FA did not deteriorate renal function in patients with diabetic nephropathy [8]. A prospective randomized study with a large number of patients would be required to fully address that question, but the present study suggested that FA is generally safe in terms of acute renal injury, even for patients with poor kidney function.

Other risk factors for CIN include diabetes and congestive heart failure [9]. Neither diabetic patients nor those with congestive heart failure showed an increase in SCr or eGFR after FA. Advanced age has been identified as a risk factor for CIN in many studies [12, 33, 34]. We did not find the association between patient age and CIN development.

The drawbacks of this study included the small number of patients and lack of long-term prospective results. This is the first study to examine CIN associated with FA, and future larger studies are required to validate our findings. Due to the retrospective nature of the present study, the comprehensive analysis on concomitant medication, especially patients’ own drugs that could have effects on renal function, could not be performed. Another limitation was that the majority of the patients included in the study were inpatients (149 of 160 patients; 93.1%). As inpatients presumably experience a higher number of comorbid conditions relative to outpatients, the incidence of CIN in everyday clinic may actually be even lower than that found in the present study. It must be noted that FA is often performed as an outpatient clinical procedure. Both cases of CIN in the present study were found in inpatients.

## Conclusions

CIN occurred in 1.3% of patients after FA. This rate was lower than reported rates after coronary angiography or contrast-enhanced CT. The use of a noniodinated agent, lower dosage, and an intravenous route of administration may account for the lower incidence of CIN associated with FA. All patients with CIN displayed normal SCr levels within 10 days without the need for hemodialysis, and did not show significant sequelae thereafter. SCr levels and eGFR remained unchanged after FA regardless of basline renal function and comorbidities. FA appeared to be generally safe in terms of acute renal injury.

## Abbreviations

CIN: Contrast-induced nephropathy
CKD: Chronic kidney disease
CT: Computed tomography
DM: Diabetes mellitus
DMN: Diabetic nephropathy
eGFR: Estimated glomerular filtration rate
FA: Fluorescein angiography
HF: Congestive heart failure
SCr: Serum creatinine
